# Tools for the Assessment of Comorbidity Burden in Rheumatoid Arthritis

**DOI:** 10.3389/fmed.2018.00039

**Published:** 2018-02-16

**Authors:** Fawad Aslam, Nasim Ahmed Khan

**Affiliations:** ^1^Division of Rheumatology, Mayo Clinic, Scottsdale, AZ, United States; ^2^Division of Rheumatology, University of Arkansas for Medical Sciences & Central Arkansas Veterans Health Care System, Little Rock, AR, United States

**Keywords:** rheumatoid arthritis, comorbidity, multimorbidy, indices, outcomes research

## Abstract

**Introduction:**

Comorbidities influence the prognosis, clinical outcomes, disease activity, and treatment response in rheumatoid arthritis (RA). RA patients have a high-comorbidity burden necessitating their study. Comorbidity indices are used to measure comorbidities and to study their impacts on different outcomes. A large number of such indices are used in clinical research. Some indices have been specifically developed in RA patients.

**Aim:**

This review aims to provide an overview of generic and specific comorbidity indices commonly used in RA research.

**Methods:**

We performed a critical literature review of comorbidity indices in RA using the PubMed database.

**Results/discussion:**

This non-systematic literature review provides an overview of generic and specific comorbidity indices commonly used in RA studies. Some of the older but commonly used comorbidity indices like the Charlson comorbidity index and the Elixhauser comorbidity measure were primarily developed to estimate mortality risk from comorbid diseases. They were not specifically developed for RA patients but have been widely used in rheumatology comorbidity measurement. Of the many comorbidity indices available, only the rheumatic disease comorbidity index (RDCI) and the multimorbidity index have been specifically developed in RA patients. The functional comorbidity index was developed to look at functional disability and has been used in RA patients considering that morbidity is more important than mortality in such patients. While there is limited data comparing these indices, available evidence seems to favor the use of RDCI as it predicts mortality, hospitalization, disability, and healthcare utilization. The choice of the index, however, depends on several factors such as the population under study, outcome of interest, and sources of data. More research is needed to study the RA-specific comorbidity measures to make evidence-based recommendations for the choice of a comorbidity measure.

## Introduction

Comorbidity has been defined as the “existence or occurrence of any additional entity during the clinical course of a patient who has the index disease under study” (1). From the research perspective, the study of comorbidities is important for several reasons: avoidance of confounding, identification of effect modification, utilization of comorbidities as predictors of outcomes or natural history, and improvement of statistical performance by converting comorbidities into a single variable (2). Awareness of comorbidities is also essential from a clinical standpoint as comorbidities influence disease activity, prognosis, medication choice, adverse effects, treatment response, patient compliance, and health care costs. Lately, increasing emphasis has been placed on the recognition, impact, and management of comorbidities prevalent in patients with rheumatic diseases (3–5).

Another concept is of multimorbidity which advocates a more holistic approach toward the patient rather than observing a patient through the prism of the index disease. Multimorbidity has been defined as “coexistence of two or more chronic diseases in the same individual” (6). This concept of multimorbidity treats all morbidities on an equal footing and does not consider some as secondary or subservient (7). Unlike the approach toward an index disease with comorbidities, where the progress is assessed through the index disease, the concept of multimorbidity places emphasis on overall patient improvement. The terms comorbidity and multimorbidity are, however, commonly used interchangeably and while cognizant of the important conceptual difference between the two, for the purpose of this article, we will use the term comorbidity, owing to its common usage. Our focus will mostly be on the study of comorbidities for research and not from a clinical perspective.

## Methods

A MEDLINE, English language restricted, search was conducted. Initial search terms of “comorbidity index rheumatology” yielded 514 citations. Subsequently, the following individual terms and their combinations: rheumatoid arthritis (RA), comorbidity, multimorbiditity, comorbidity indices, and rheumatology were employed. Abstracts were reviewed to select articles relevant to this non-systematic review. Further pertinent articles were identified from the bibliography of the selected articles and were used to guide this review. Indices were selected for this review if they were the standard indices used in general comorbidity research, looked at functional status, and/or were specifically developed for studying RA patients.

## Results/Discussion

### RA and Comorbidities

Comorbidity is the norm rather than the exception in RA (8). Nearly, a third of patients have at least on comorbidity at the onset of RA with eventual involvement of nearly 80% patients during follow-up (4, 9). A patient with established RA has on average two comorbidities (10). The study and awareness of comorbidities in RA is very important from both a research and clinical perspective.

Comorbidities have a greater influence on mortality in RA patients than the shared epitope, the presence of erosions or a positive rheumatoid factor (11). Premature death in RA is attributable to the presence of such serious comorbidities, suboptimal care of these coexisting diseases, and the inflammatory milieu that propagates these comorbidities (12). Comorbidities result in increased healthcare cost, functional disability, poorer quality of life, and treatment interference in addition to the excess mortality. For, e.g., cardiovascular diseases (CVD) lead to excess mortality while depression produces disability (10). Comorbidities have a major negative impact on quality of life, causing functional disability, independent of disease activity (4, 10, 13).

Undertreatment of comorbidities is a major concern in RA patients (5, 14). Patients with comorbidities tend to get less aggressive treatment of RA despite having higher RA disease activity (8). The presence of each additional comorbidity reduces the odds of remission by 28% (15, 16). Current disease activity measures include patient reported outcomes. These patient reports are influenced by comorbidities leading to a poorer response with increasing comorbidities (16–18). Remission or low-disease activity may never be achieved if the patient global or other patient general health outcomes, originating from these comorbidities, remain high. The reversible and irreversible aspect of structural damage in RA (19) and the interplay of comorbidities must be considered by the practicing clinician when striving for remission or low-disease activity. The focus on RA disease activity control must not be at the expense of comorbidity management. Comorbidities are important from a causal aspect as well. The excess risk of CVD mortality in RA that is independent of traditional risk factors was identified from the study of comorbidities (20).

### Measuring Comorbidities

Several factors have to be taken into consideration when measuring comorbidity. First and foremost is deciding which diseases to measure. All the comorbidities could be included or a selection made. If only selected comorbidities are studied, their inclusion should be based on study pertinent criteria. After identification of comorbidities, the researcher needs to determine if the included diseases are treated as equal or assigned different weightage, e.g., the implications of having a cataract are different from that of CVD on mortality. The selection of comorbidities depends on the research question being asked, the population being studied, and the preexisting knowledge about these comorbidities. The question of the severity of the comorbidity is also important due to differing consequences, e.g., stages of chronic kidney disease. The choice of comorbidities may be different if studying inpatient mortality after a surgery versus long-term disability from a rheumatic condition. The selection of comorbidities is also governed by the source of the information for the comorbidity data, e.g., self-report, chart review, administrative database, pharmacy database, or a combination of these.

The simplest way to measure comorbidity is a disease count. It is a convenient method but can become cumbersome as the number of comorbidities increase, can suffer from lack of specificity to the research question and treats every disease equally. Comparisons between populations would be difficult with a simple disease count.

Indices are used to circumvent the aforementioned problems. An index is a composite outcome as a single number which includes specific diseases that may or may not be weighted. Use of an index allows comparisons among populations as the comorbidities being studied are similar (21). A large number of comorbidity indices have been developed and used across populations, from different data sources, and for studying different outcomes. It is beyond the scope of this article to discuss all of them. Table 1 gives a summary overview of the indices discussed in this review. There is, however, no gold standard comorbidity index. Some data suggest that while helpful due to their ease of use, time and cost-effectiveness, comorbidity indices provide only a limited ability to mitigate confounding (22) and their adjustment for confounding over using age alone as a confounder is only modest (23). On the contrary, data also show that age and gender alone have limited ability to predict mortality irrespective of the comorbidities studied and the index used (24). In clinical medicine, the utility of comorbidity indices is limited (10). Nevertheless, they are currently the most viable option for the study of comorbidities and their comparison across subjects.

**Table 1:** A comparison of major comorbidity indices.

IndexFeature	Charlson comorbidity index^a^	Elixhauser comorbidity measure^a^	Functional comorbidity index	Multimorbidity index	Rheumatic disease comorbidity index
Number of diseases^b^	19	30	18	40	11
Development year	1987	1998	2005	2015	2007
Original study population	Medical inpatients and breast cancer patients	Medical inpatients	General population and spine center patients	Rheumatoid arthritis patients	Rheumatoid arthritis, lupus, osteoarthritis, and fibromyalgia patients
Original outcome studied	One-year mortality	Length of stay, hospital costs, and hospital mortality	Physical function	Health-related quality of life	Mortality, hospitalization, disability, and costs
Administrative database use	Yes	Yes	Yes	Yes	Yes
Self-report use	Yes	No	No	No	Yes
Weightage versions	Weighted	Weighted and unweighted	Unweighted	Weighted and unweighted	Weighted
Fibromyalgia included	No	No	No	No	No

*^a^Includes adaptations*.

*^b^Including different severities of a single disease*.

### Charlson Comorbidity Index (CCI)

The CCI is the most widely used comorbidity index (25). It was published in 1987 to predict 1-year mortality in a cohort of patients admitted to a medical service and then validated in a cohort of breast cancer patients. It has 19 conditions (16 diseases of which 3 are stratified according to severity) which are weighted differently based on their mortality association and then are added to give the index score (Table S1 in Supplementary Material). The final score can vary from 0 to 33. While originally developed to prospectively predict 1-year mortality among patients being considered for breast cancer clinical trials, it has been shown to be predictive of other outcomes such as inhospital mortality, length of hospital stay, readmission rate, functional decline, and healthcare utilization (26–28). In a population with low morbidity and high mortality, the CCI has had various adaptations and has performed well. It has been adapted for use in administrative databases using both International Classification of Disease, Ninth Edition, Clinical Modification (ICD-9-CM), International Classification of Disease, Tenth Edition (ICD-10) as well as ICD-9 without clinical modification (29–31) classification systems. It has also been validated as a self-report tool for comorbidities (26–28). Although the agreement between the self-report CCI and the administrative CCI was moderate, the two indices showed similar predictive validity for outcomes such as functional decline and health care utilization (29, 30). Since age is a major determinant of mortality, a combined age-comorbidity CCI has been validated where each decade above the age of 50 scores an additional point, e.g., a 50- to 59-year-old subject will get a score of one based on age alone (32). CCI has been used to predict disability and functional status although that was not its original intention. It has not been validated for the health-related quality of life (HRQoL) outcome. CCI has been used in rheumatology and has shown that comorbidity leads to increased disability in RA patients (13, 33). CCI has been shown to be a significant independent predictor of mortality in a population-based prevalence cohorts with RA and osteoarthritis (OA) (34). Because of its widespread use and validity, it is still the preferred index of many researchers.

For RA patients, CCI, however, does not account for important RA comorbidities like hypertension, osteoporosis, OA, obesity, and fibromyalgia which can have a significant impact from a disability, disease control, and health cost perspective. Fibromyalgia can lead to failure of RA disease remission (35). CCI also does not account for psychiatric conditions such as depression and anxiety which have been shown to be associated with higher disease activity, reduced likelihood of remission, increased functional disability, and increased discontinuation of biologic treatment (36–38). CCI was designed to predict mortality but the latter, in contrast to breast cancer patients in the original study, is less important in rheumatology when compared with physical function, cost, and morbidity (39).

### Elixhauser Comorbidity Measure (ECM)

The ECM was developed for use in administrative inpatient databases to predict hospital charges, length of stay, and in-hospital mortality using records of 1,779,167 patients in Statewide Inpatient Database of California for the year 1992. It is one of the most widely used indices in comorbidity research. It comprises 30 dichotomous comorbidities (Table S2 in Supplementary Material) but without weighting and thus without a single score (40). It does include conditions like obesity, mental disorders, alcoholism, and hypertension that had been excluded from some other indices. It does not include OA but includes RA and collagen vascular diseases as a single category. The comorbidity list needs to be revised to explicitly exclude comorbidities related to the principal diagnosis under study. A successful modification was tested for ICD-9-CM and ICD-10 (30). Of note, Elixhauser et al. chose to retain comorbidities as separate, independent measures, and recommended against using an index because different comorbidities produce different outcomes and treatments depending on the patient population. Nevertheless, alternative approaches to obtain a single score either using a simple summation of all present comorbidity (one point per comorbidity) to obtain total ECM score or assigning weights to different comorbidities have been used (39, 41). The former was used in OA patients to predict health service utilization. There is scant literature on assigning weightage in ECM scores and is only available in the context of mortality data (41). No rheumatology specific data are available. Of note, ECM tends to outperform CCI in predicting mortality (42).

### Functional Comorbidity Index (FCI)

Functional comorbidity index was developed with the intention to look at physical disability in the general population and to circumvent indices designed for predicting mortality and other measures (43). A pool of 40 comorbidities as potential predictors was generated from systematic literature review and focus groups of patients and clinicians. An 18-item index from this pool was developed using two databases: a cross-sectional, simple random sample of 9,426 Canadian adults, and a sample of 28,349 US adults seeking treatment for spinal diseases. It includes conditions like arthritis (RA and OA), degenerative disc disease (including chronic severe low-back pain), visual compromise, and osteoporosis that are important from a physical function approach (Table S3 in Supplementary Material). A simple count and a weighted count were developed, but being grossly similar, the simple count was encouraged owing to its ease of use. FCI is scored from 0 to 18. Severity of disease was not rated and while acknowledging the impact of severity, the authors cited the variability of severity ratings and problems with documentation accuracy as reasons enough to forgo severity rating. FCI, to its credit, explains more of the variance in physical function as measured by short form 36 (SF-36) physical function subscale (29%) than the CCI explains variance in mortality (19.5%). FCI is a better predictor of general health status than CCI (43). FCI is best suited for assessing physical disability and function and not the ideal tool for mortality assessment. Since these are so relevant to RA, it was included for this review.

### Multimorbidity Index (MMI)

Multimorbidity index is a validated index comprising 40 conditions based on HRQoL, as assessed by EuroQol-five dimensional (EQ-5D), in the RA population (44). Unlike several other indices, where the selection of comorbidities is empiric or based on prevalence rates (45), the comorbidities in the MMI are based on systematic reviews or recommendations of the National Health Service of Scotland (Table S4 in Supplementary Material). It has a simple count-based measure and a weighted measure. Not much improvement was gained by weighing, hence a simple count-based MMI is more feasible. It does not take disease severity into consideration. RA activity was not considered either because it lacked a significant impact on the conclusion. Certain factors like fatigue, socioeconomic status, and pain are not included. In the validation studies, MMI better explained the variance in EQ-5D than the CCI (44). Higher MMI score was shown to negatively affect achievement of therapeutic goal of remission or low-disease activity in a prospective RA cohort 1 year after starting DMARD (15). Collecting data on 40 comorbidities is not easy but in their validation cohort, in which data on all 40 comorbidities was unavailable, the MMI still performed well. Please note that there are several other multimorbidity indices that are used for different conditions. The MMI referenced here was developed specifically for RA patients.

### Rheumatic Disease Comorbidity Index (RDCI)

Rheumatic disease comorbidity index is weighted and was developed based on self-report from patients with RA, OA, lupus, and fibromyalgia (10, 46). It is rated from 0 to 9 and comprises 11 comorbid conditions including fracture, depression, and peptic ulcer disease (Table S5 in Supplementary Material). It was subsequently validated in a clinic self-report data set as well as assessed for ability to predict physical disability as well as death in an administrative data set of RA patients (46). The study utilized three models: bare, administrative, and clinical. The administrative model used information on visit frequency, weight, prednisone, and methotrexate use. The clinical model further assessed erythrocyte sedimentation rate, rheumatoid factor, rheumatoid nodules, and routine assessment of patient index data 3 as a disease activity scale. Overall, the clinical model performed the best but all models had similar ranking across similar indices. FCI was the best predictor for physical disability followed by RDCI and unweighted ECM. RDCI was the best predictor for mortality followed by unweighted ECM and modified CCI. FCI was least helpful in assessing mortality. Overall RDCI and unweighted ECM performed well for both physical disability and mortality (46). The authors preferred RDCI as unlike ECM which can only be used in administrative database, RDCI can also be used with patient report of data. RDCI had the same predictive value as ECM but only requires 11 versus 30 comorbidities and thus easier to use. RDCI has the advantage of not over adjusting for the index disease as it does not have a musculoskeletal comorbidity category (47). A modified version of RDCI was tested in gout patients and the original RDCI was validated for use in gout patients as well (47).

### Comparison of Indices

Majority of the studies show that the ECM and its adaptations perform better than CCI and its modifications at predicting mortality (48–50). The CCI, however, continues to be used perhaps because of its widespread use across multiple conditions and the ease of collecting data on 19 variables versus the 30 in ECM (21). The paper by Yurkovich et al. provides an excellent systematic overview of different indices derived from administrative health data and their adaptations across disparate medical conditions (42). Compared with CCI (18%), the FCI explained significantly more (29%) variation in physical function (43). FCI has also been found to be a more robust predictor of general health status in patients with sleep apnea, chronic rhinosinusitis, and acute lung injury (51–53). Weighted MMI showed the best correlation with HRQoL, fatigue, and physical function followed by FCI and CCI (44). Count-based MMI performed less well than the weighted version but still performed better than the CCI. Both versions predict HRQoL at 1 year. FCI was the best predictor for physical disability followed by RDCI and unweighted ECM in RA patients (46). RDCI was the best predictor for mortality followed by unweighted ECM and modified CCI (46). FCI was least helpful in assessing mortality. Overall RDCI and unweighted ECM performed well for both physical disability and mortality (46). One recent study has compared modified CCI, RDCI, and FCI in RA patients (54). Clinical outcomes like hospitalization, health assessment questionnaire (HAQ), and mental and physical components of SF-36 were assessed. All three indices were associated with each outcome and differences in their performance were moderate. The RDCI was the simplest to use. The RDCI and FCI performed better on predicting hospitalization, HAQ, and SF-36 compared with CCI. The study did not assess mortality.

### Choosing an Index

The choice of an index is dictated by several factors as there is no gold standard. Index performance varies based on the index used, the outcome being studied, and the population. An index is also affected by the limitations of data sources, e.g., administrative database versus self-report, e.g., ECM cannot be used with the latter. Indices also do not allow study of individual comorbidities and their influence on the outcomes of interest. The impact of individual comorbidity on the primary disease of interest may be variable. It has been shown that cardiovascular comorbidities, in particular hyperlipidemia, diabetes, ischemic heart disease, and obesity, were associated with measures of RA disease activity more than any other comorbidity (55). The quality and completeness of data will determine the number of comorbidities that are available and thus selecting an instrument that can make efficient use of the available information, e.g., RDCI only requires 11 comorbidities whereas MMI works with 40. The research question at hand will dictate the use of a general mortality index, a function index, or an index that will provide information for both outcomes, e.g., if multiple outcomes are being studies RDCI may be better as it looks at mortality, hospitalizations, disability, and health care costs. On the contrary, if disability is the only outcome of interest FCI may be used. The study population determines if a questionnaire-based index will be required and how comparable it is to the population in which the index was validated. Utilizing a “lookback” period where data from the previous 1–2 years is analyzed and modeled into the projections has a better explanatory power (56). There have been publications of empiric indices, which are study and population specific, which tend to have better predictive ability (57, 58) but create difficulties when comparing studies.

The indices developed specifically for RA patients are newer when compared with other indices. Therefore, there is scant literature comparing them to other indices. Many research studies in rheumatology still use the older indices, e.g., CCI to determine comorbidity burden. Of these older indices, ECM tends to outperform CCI in predicting mortality (42). As studies start using these rheumatology-specific indices, a better measure of their comparative performance will be forthcoming.

## Sources of Data

### Administrative Databases

These are the most widely used. Using codes to identify diseases allows utilizing large patient databases in a time efficient and cost effective manner. However, databases can suffer from omissions, problems with country-specific coding, coding bias, incorrect coding, and lack a measure of disease chronicity (26, 42).

### Self-Report

Self-report is good for serious and surgical diagnoses (59). It is the instrument of choice when medical records are sparse or unavailable. Language issues, cultural concepts of disease, and subject recall can affect data obtained from self-report. Particularly for RA, patients overestimate RA as they do not differentiate RA from other arthritic conditions (60). There is variable concordance between self-report and administrative databases ranging from similar to poor (26, 28, 61, 62). RDCI and CCI have been used with self-report data. Self-report is encouraged only when chart data are not easily available. With CCI in RA, use of self-report is not recommended (63).

### Chart Review

Chart review requires the services of an abstracter to comb through all the medical records of study subjects. This can be expensive and time consuming. The results are dependent on the completeness of the records and can suffer from problems of omissions. They are impractical for large population studies or complicated patients where the amount of medical chart data is extensive. Chart review, however, tends to perform better than administrative databases (64). It may be appropriate for studying small populations and asymptomatic diseases (2). Another source of data is pharmacy based on prescription use which we have not discussed.

## Conclusion

The study of comorbidities is critical in research involving RA patients due to their high- comorbidity burden. The best way to do so is through the use of comorbidity indices. There is no gold standard comorbidity index but recently there has been research leading to development of rheumatology and RA-specific indices. The RDCI and MMI have been developed for use specifically in rheumatology patients and have been tested in RA patients. It is to be seen what impact, if any, the inclusion of conditions like fibromyalgia and infection history/infection risk would have on rheumatology-specific indices. Further studies are needed to compare these indices in rheumatology patients. Identification of an optimal index will then allow comparability of comorbidities across studies and their influence on clinical and treatment outcomes. At present, RDCI offers the advantages of being simple and capable of utilization with questionnaires as well as administrative databases.

## Author Contributions

Planning the topic, outlay of manuscript, literature review, drafting manuscript, and revisions for the submitted version.

## Conflict of Interest Statement

The authors declare that the research was conducted in the absence of any commercial or financial relationships that could be construed as a potential conflict of interest.
