# Supplementary material for: Tools for the Assessment of Comorbidity Burden in Rheumatoid Arthritis
Source: Front Med (Lausanne). 2018 Feb 16;5:39. doi: 10.3389/fmed.2018.00039 (PMC5820312; doi:10.3389/fmed.2018.00039)
Supplement: Supplementary file 1 [file table_1.docx]

Supplementary Table 1. Charlson Comorbidity Index

| **Comorbid condition** | **Points** |
| --- | --- |
| Myocardial Infarction | 1 |
| Congestive Heart Failure | 1 |
| Peripheral Vascular disease | 1 |
| Cerebrovascular disease | 1 |
| Dementia | 1 |
| COPD | 1 |
| Connective Tissue disease | 1 |
| Peptic Ulcer disease | 1 |
| Diabetes Mellitus | 1 point if uncomplicated  2 points if complicated |
| Moderate to severe CKD | 2 |
| Hemiplegia | 2 |
| Leukemia | 2 |
| Malignant Lymphoma | 2 |
| Solid Tumour | 2 points  6 points if metastatic |
| Liver disease | 1 point if mild  3 points if moderate to severe |
| AIDS | 6 points |

Adapted from: Charlson ME, Pompei P, Ales KL, MacKenzie CR. A new method of classifying prognostic comorbidity in longitudinal studies: development and validation. *J Chronic Dis* (1987) 40(5):373-83. PubMed PMID: 3558716.

COPD: chronic obstructive pulmonary disease; CKD: chronic kidney disease; AIDS: acquired immunodeficiency syndrome.
