# Supplementary material for: Tools for the Assessment of Comorbidity Burden in Rheumatoid Arthritis
Source: Front Med (Lausanne). 2018 Feb 16;5:39. doi: 10.3389/fmed.2018.00039 (PMC5820312; doi:10.3389/fmed.2018.00039)
Supplement: Supplementary file 2 [file table_2.docx]

Supplementary Table 2. Elixhauser’s Comorbidity measure

| **Comorbid condition** |
| --- |
| Congestive heart failure |
| Cardiac arrhythmias |
| Valvular disease |
| Pulmonary circulation disorders |
| Peripheral vascular disorders |
| Hypertension |
| Paralysis |
| Other neurological disorders |
| COPD |
| Diabetes uncomplicated |
| Diabetes complicated |
| Hypothyroidism |
| Renal Failure |
| Liver disease |
| Peptic ulcer excluding bleeding |
| AIDS |
| Lymphoma |
| Metastatic cancer |
| Solid tumor without metastasis |
| Rheumatoid arthritis/collagen vascular diseases |
| Coagulopathy |
| Obesity |
| Weight loss |
| Fluid and electrolyte disorders |
| Blood loss anemia |
| Deficiency anemia |
| Alcohol abuse |
| Drug abuse |
| Psychosis |
| Depression |

Adapted from: Elixhauser A, Steiner C, Harris DR, Coffey RM. Comorbidity measures for use with administrative data. *Med Care* (1998) 36(1):8-27. PubMed PMID: 9431328.
