# Supplementary material for: Tools for the Assessment of Comorbidity Burden in Rheumatoid Arthritis
Source: Front Med (Lausanne). 2018 Feb 16;5:39. doi: 10.3389/fmed.2018.00039 (PMC5820312; doi:10.3389/fmed.2018.00039)
Supplement: Supplementary file 3 [file table_3.docx]

Supplementary Table 3. Functional comorbidity index

| **Comorbid condition** |
| --- |
| Arthritis (rheumatoid or osteoarthritis) |
| Osteoporosis |
| Asthma |
| COPD/Emphysema/ARDS |
| Angina |
| Congestive heart failure (or heart disease) |
| Heart attack (myocardial infarct) |
| Neurological disorder (such as multiple sclerosis or Parkinson’s) |
| Stroke or TIA |
| Peripheral vascular disease |
| Diabetes types I and II |
| Upper gastrointestinal disease (ulcer, reflux, hernia) |
| Depression |
| Anxiety or panic disorders |
| Hearing impairment (very hard of hearing, even with hearing aids) |
| Visual impairment (such as cataract, glaucoma, macular degeneration) |
| Degenerative disc disease (spinal stenosis, back disease or severe chronic back pain) |
| Obesity and/or BMI > 30 |

Adapted from: Groll DL, To T, Bombardier C, Wright JG. The development of a comorbidity index with physical function as the outcome. *J Clin Epidemiol* (2005) 58(6):595-602. doi: 10.1016/j.jclinepi.2004.10.018. PubMed PMID: 15878473.
