# Supplementary material for: Tools for the Assessment of Comorbidity Burden in Rheumatoid Arthritis
Source: Front Med (Lausanne). 2018 Feb 16;5:39. doi: 10.3389/fmed.2018.00039 (PMC5820312; doi:10.3389/fmed.2018.00039)
Supplement: Supplementary file 4 [file table_4.docx]

Supplementary Table 4. Multimorbidity index (MMI)

| **Comorbid condition** | **Assigned weight*** |
| --- | --- |
| Glaucoma | 0.5 |
| Irritable bowel syndrome | 0.5 |
| Schizophrenia, bipolar disorder | 0.5 |
| Learning disability | 0.5 |
| Anorexia/bulimia | 0.5 |
| Migraine | 0.5 |
| Prostate disorders | 0.5 |
| Diverticulitis | 0.5 |
| Chronic sinusitis | 0.5 |
| Hypertension | 0.5 |
| Cancer | 0.5 |
| Diabetes | 0.5 |
| Atrial fibrillation | 0.5 |
| Constipation | 0.5 |
| Multiple sclerosis | 1 |
| Substance misuse | 1 |
| Osteoporosis | 1 |
| Psoriasis eczema | 1 |
| Coronary heart disease | 1 |
| Hearing loss | 1 |
| Stroke/transient ischemic attack (TIA) | 2 |
| Peripheral vessel disease | 2 |
| Chronic kidney disease | 2 |
| Inflammatory bowel disease | 3 |
| Thyroid disorders | 3 |
| Asthma | 3 |
| Obesity | 4 |
| Chronic liver disease | 5 |
| Heart failure | 5 |
| Bronchiectasis | 5 |
| Depression | 6 |
| Anxiety/neurotic disorders | 8 |
| Alcohol problems | 9 |
| Blind or low vision | 10 |
| Parkinson | 10 |
| Dyspepsia | 10 |
| Chronic obstructive pulmonary disease (COPD) | 10 |
| Hepatitis (viral) | 10 |
| Epilepsy | 20 |
| Dementia | 20 |

Adapted from: Radner H, Yoshida K, Mjaavatten MD, Aletaha D, Frits M, Lu B, et al. Development of a multimorbidity index: Impact on quality of life using a rheumatoid arthritis cohort. *Semin Arthritis Rheum* (2015) 45(2):167-73. doi: 10.1016/j.semarthrit.2015.06.010. PubMed PMID: 26209213.

*MMI score may be calculated on numerical count of comorbid conditions (MMI.count) or using the assigned weight for each comorbid condition (MMI.weight).
