# Supplementary material for: Tools for the Assessment of Comorbidity Burden in Rheumatoid Arthritis
Source: Front Med (Lausanne). 2018 Feb 16;5:39. doi: 10.3389/fmed.2018.00039 (PMC5820312; doi:10.3389/fmed.2018.00039)
Supplement: Supplementary file 5 [file table_5.docx]

Supplementary Table 5. Rheumatic Disease Comorbidity Index

| **Conditions** | Lung disease  Heart Attack  Other CV disease  Stroke  Hypertension  Diabetes  Fracture (of hip/spine/leg)  Depression  Cancer  Gastrointestinal ulcer  Other stomach problems  Lung disease |
| --- | --- |
| **Calculation** | 2 X lung disease + [2 X (heart attack, other CV disease, OR stroke) OR 1 X hypertension] + Fracture + Depression + Diabetes + Cancer + (ulcer OR stomach problem) |

Adapted from: England BR, Sayles H, Mikuls TR, Johnson DS, Michaud K. Validation of the rheumatic disease comorbidity index. *Arthritis Care Res (Hoboken)* (2015) 67(6):865-72. doi: 10.1002/acr.22456. PubMed PMID: 25186344.
